# Maleamic Acid as an Organic Anode Material in Lithium-Ion Batteries

**DOI:** 10.3390/polym12051109

**Published:** 2020-05-13

**Authors:** Berhanemeskel Atsbeha Kahsay, Fu-Ming Wang, Alem Gebrelibanos Hailu, Chia-Hung Su

**Affiliations:** 1 Graduate Institute of Applied Science and Technology, National Taiwan University of Science and Technology, Taipei 106, Taiwan; alemachd@gmail.com (B.A.K.); alemg2003@gmail.com (A.G.H.); 2 Sustainable Energy Center, National Taiwan University of Science and Technology, Taipei 106, Taiwan; 3 Department of Chemical Engineering, Chung Yuan Christian University, Taoyuan 320, Taiwan; 4 R&D Center for Membrane Technology, Chung Yuan Christian University, Taoyuan 320, Taiwan; 5 Graduate School of Biochemical Engineering, Ming Chi University of Technology, New-Taipei City 243, Taiwan; chsu@mail.mcut.edu.tw

**Keywords:** maleamic acid, carbon black, lithium-ion batteries, organic anode

## Abstract

Low-molecular-weight carbonyl-containing compounds are considered beneficial energy storage materials in alkali metal-ion/alkaline earth metal-ion secondary batteries owing to the ease of their synthesis, low cost, rapid kinetics, and high theoretical energy density. This study aims to prepare a novel carbonyl compound containing a maleamic acid (MA) backbone as a material with carbon black to a new MA anode electrode for a lithium-ion battery. MA was subjected to attenuated total reflection-Fourier-transform infrared spectroscopy, and its morphology was assessed through scanning electron microscopy, followed by differential scanning calorimetry to determine its thermal stability. Thereafter, the electrochemical properties of MA were investigated in coin cells (2032-type) containing Li metal as a reference electrode. The MA anode electrode delivered a high reversible capacity of about 685 mAh g^−1^ in the first cycle and a higher rate capability than that of the pristine carbon black electrode. Energy bandgap analysis, electrochemical impedance, and X-ray photoelectron spectroscopy revealed that MA significantly reduces cell impedance by reforming its chemical structure into new nitrogen-based highly ionic diffusion compounds. This combination of a new MA anode electrode with MA and carbon black can increase the performance of the lithium-ion battery, and MA majorly outweighs transitional carbon black.

## 1. Introduction

Lithium-ion batteries (LIBs) are the most extensively used energy sources for portable electronic devices, automatic machines, electric vehicles, and power tools owing to their high energy density, long lifecycle, and high voltage outputs [1,2]. The conventional graphite anode in LIBs has a low working potential, flat potential profile, and low cost. However, graphite only provides 300–340 mAh g^−1^ of energy and presents a high risk of lithium plating issues. Furthermore, graphite is not suitable for sodium-ion batteries because it forms unstable intercalated graphite [3]. Lithium metal has recently been used actively as anode material owing to its high specific energy (3860 mAh g^−1^), low negative electrochemical potential, and low weight. Unfortunately, lithium metal is active and has high safety concerns because of intractable dendrite growth during electrochemical lithium plating [4]. Furthermore, the low abundance of lithium metal in nature is another industrial issue.

Organic electrode materials, including conductive polymer [5,6,7], organosulfur [8], organic free radical [9], and carbonyl [10,11,12,13,14] compounds have been considered promising anode materials for alkali metal-ion/alkaline earth metal-ion batteries, such as Li^+^, Mg^2+^, Na^+^, Ca^2+^, and K^+^, because of the ease of their synthesis, high renewability, low cost, structural diversity, environmental compatibility, and high theoretical capacities. Among several organic materials, carbonyl compounds have been intensively studied because of their high theoretical capacity and rapid reaction kinetics [15]. However, state-of-the-art organic electrodes still have low electronic conductivity and high solubility in aprotic solvents, resulting in a marked reduction in capacity, an unstable structure [16], high polarization potential owing to poor electronic conductivity [10,17], and the absence of distinct plateau regions in voltage profiles [10,17,18]. Several strategies have been assessed to alleviate issues regarding capacity reduction and short cycle life, such as the use of solvents [19,20], salt [12,21], electronically conductive carbon-based composite [22,23,24], and molecular weight modifications [6,25]. Low-molecular-weight compounds usually have a short cycle life owing to their high solubility in aprotic electrolytes. Armand et al. used a combination of aliphatic carboxylate and trans, trans-muconate as an anode material, and it delivered 205 mAh g^−1^ in the first cycle and 125 mAh g^−1^ after 80 cycles at 0.05 C [23]. Fedele et al. used di-lithium 2,6-naphthalene dicarboxylate, yielding 205 mAh g^−1^ in the initial cycle and decreasing to 91 mAh g^−1^ after 50 cycles at 0.05 C [26]. Chen et al. used anhydrous di-lithium rhodizonate as an organic electrode for lithium-ion batteries, yielding 580 mAh g^−1^ in the first cycle and decreasing to 280 mAh g^−1^ after 14 cycles at 0.025 C [27]. Low-molecular-weight aliphatic compounds generally cannot easily store lithium ions owing to the low storage potential of carbonyl units [28,29,30]. The aforementioned reports indicate that organic carbonyl compounds are not stable during electrochemical reactions, thus limiting their future applications.

This study aimed to investigate the redox properties of maleamic acid (MA) as a new MA anode material for LIBs.

## 2. Experimental

### 2.1. Materials

MA (98%) was purchased from Tokyo Chemical Industry (CHUO-KU, Tokyo, Japan) and directly used as organic anode material without further modification. Super-P (SP) (99%) was purchased from Sigma-Aldrich (St. Louis, MO, USA).

### 2.2. Material Characterization

Attenuated total reflection-Fourier transform infrared spectroscopy (ATR-FTIR) was performed using an optics spectrometer (FT/IR-6700, JASCO, Osaka, Japan) at 4000−400 cm^−1^. X-ray diffraction pattern (XRD) measurement was performed using an X-ray diffractometer (D_2_ phaser, Bruker, Karlsruhe, Germany) with Cu Kα radiation with a range from 2θ values of 10° to 70° with the increment of 0.5° and scanning rate of 5° per minute. MA morphology was assessed through scanning electron microscopy (SEM) after Pt-coating the electrode in a glove box at an accelerating voltage of 5 kV using the LEO-1530 microscope (Zurich, Switzerland). Differential scanning calorimetry (DSC) was performed in a nitrogen environment from 30 to 400 °C at a heating rate of 10 °C min^−1^ using a Perkin-Elmer DSC-4000 analyzer, Akron, OH, USA. The energy level was determined using a density functional theory (DFT) method that combines the Becke-3-parameter-Lee–Yang–Parr hybrid functional with the basis set 6-31G (d,p). X-ray photoelectron spectroscopy (XPS) was conducted after Pt-coating the electrode in a glove box at an accelerating voltage of 15 kV using a PHI 1600S spectrometer (Japan) to investigate the structural information of the electrodes in the pristine state and after electrochemical cycles using Al Kα sources of radiation (1486.6 eV). The coin cells of MA and SP electrodes after the cyclability test were disassembled in an argon-filled glove box and collected for XPS measurements.

### 2.3. Assessment of Electrochemical Properties

Electrochemical properties were investigated in CR2032-type coin cells comprising the working electrode (MA was homogenized with SP) and lithium metal as a counter/reference electrode. The MA electrode was generated by mixing 60 wt % MA, 30 wt % SP, and 10 wt % carboxymethyl cellulose (CMC) binder. The SP electrode was generated by casting the slurry containing 90 wt % SP and 10% CMC. The mass loading of both active materials (MA and SP) have similar loadings about 1.6 mg cm^−2^. The electrolyte comprised 1 M LiPF_6_ in ethylene carbonate, propylene carbonate, and diethyl carbonate (3:2:5 by *v/v* ratio). Galvanostatic cycling was performed using a BAT-750B battery tester at 3.0–0.0 V (vs. Li/Li^+^). Cyclic voltammetry (CV) was performed using Biologic VMP3 at a scan rate of 0.1 mV s^−1^ under the same potential window. Electrochemical impedance spectroscopy (EIS) was performed with VMP3 at 1–10 mHz at an amplitude of 5 mV.

## 3. Results and Discussion

**Figure 1:**
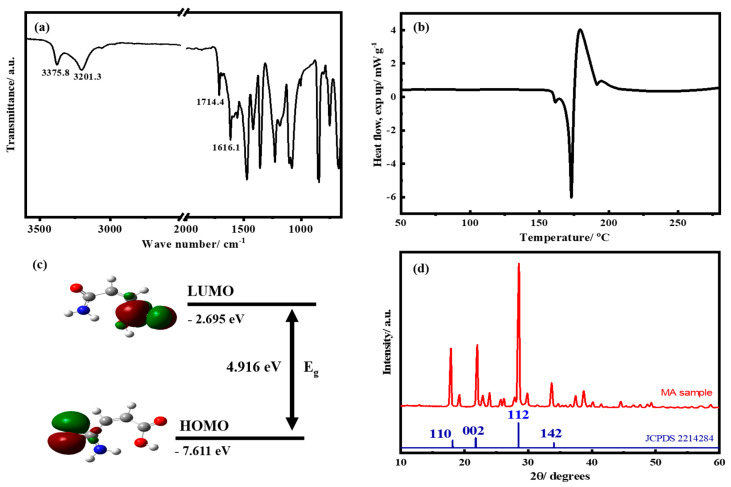
(**a**) The attenuated total reflection (ATR)-FTIR spectra, (**b**) the differential scanning calorimetry (DSC) analysis, (**c**) the calculation of HOMO/LUMO energy level, and (**d**) the XRD pattern of the maleamic acid (MA) material.

Figure 1a shows the attenuated total reflection-Fourier-transform infrared spectroscopy (ATR-FTIR) spectrum of pristine MA. The carboxylic and amide groups of MA were detected at 3375.8 and 3201.3 cm^–1^. Two absorption peaks at 1714.4 and 1686.4 cm^–1^ were obtained for the C=O component of carboxylic and the C=O of amide groups, respectively. Absorption peaks at 1556 and 1357 cm^−1^ represent carboxyl asymmetric and symmetric vibrational modes, respectively. DSC analysis was performed to evaluate the thermal behaviors of MA (Figure 1b). Consequently, the melting point of MA was 175 °C without any thermal decomposition below 300 °C. To determine the average redox potential and energy bandgap of MA, the molecule orbital energy levels were determined through DFT calculations. The lower the lowest unoccupied molecular orbital (LUMO) of the molecule, the higher the reduction potential and electron affinity [31]. Figure 1c shows that the LUMO of MA was −2.695 eV, which was markedly lower than that of carbon nanotubes (−2.047 eV) [32] and Si (−0.145 eV). Figure 1d shows the XRD patterns of MA; these spectra display major peaks at 18.12°, 21.76°, 28.46°, and 34.03°, attributed to the ($\bar{1}\bar{1}$0), (00$\bar{2}$), ($\bar{1}\bar{1}\bar{2}$), and ($\bar{1}\bar{4}\bar{2}$) planes (COD 22142284), respectively, with an orthorhombic crystalline structure.

**Figure 2:**
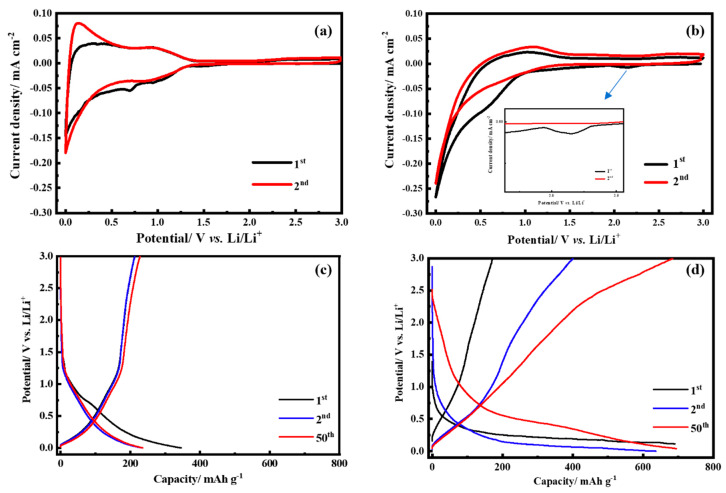
The cyclic voltammetry (CV) measurements of (**a**) Super-P (SP) and (**b**) MA electrodes at a scan rate of 0.1 mV s^−1^. The charge and discharge profiles of (**c**) the SP and (**d**) the MA electrodes at 0.1 C at the first and the 50th cycle.

Figure 2a,b displays the results of CV analysis for the SP and the MA electrodes. As shown in Figure 2a, the SP electrode containing SP only presents a common redox behavior [33]. A minor reduction reaction at 0.79 V and an irreversible de-lithiation reaction at 0.1 V occurred in the first cycle, corresponding to the irreversible formation of the solid electrolyte interphase (SEI) [34]. In subsequent cycles, CV curves revealed indistinguishable results, indicating adequate electrochemical stability of SP. As shown in Figure 2b, the MA electrode was completely different from the SP electrode. A broader reduction reaction at 2.13 V occurred in the first cycle and was obliterated in the subsequent cycle. This result suggests that electrolyte decomposition may be used to initiate at the MA electrode owing to the electrochemical replacement reaction between the proton in the carbonyl group and lithium ions, thus the redox reaction of the MA structure. A similar electrochemical reaction has been previously observed with other carbonyl-containing organic materials including maleic acid [30], ellagic acid [35], sodium terephthalate [36], and 2,5-dihydroxyterephthalic acid [37]. Furthermore, the SEI was formed at 1.22 to 0.75 V. In the following second and third cycles, CV displayed no differences, indicating the superior reversibility and accessibility of the composite electrode. However, the de-lithiation reaction at the MA electrode was altered upon the redox reaction of the MA, occurring instead at a higher potential range of approximately 0.5 to 1.0 V. Figure 2c shows the charge–discharge curves for the SP electrode. The initial discharge capacity of the SP electrode delivers 212.2 mAh g^−^^1^ at 0.1 C with a coulombic efficiency of approximately 61.4% and achieves to 99.5% at further cycles. Figure 2d shows that the MA electrode delivers only at 171.9 and 401.0 mAh g^−1^ at 0.1 C in the first and second cycles, yielding a low coulombic efficiency of 24.9 and 63.0%, respectively. The low redox efficiency at the first cycle may be attributed to the redox reaction of the MA. However, the redox reaction of the MA was not completed during the first redox reaction and the capacity gradually increased to 685.4 mAh g^−1^ (98.6% coulombic efficiency) at the 50th cycle. These results indicate that the redox reaction of the MA occurs gradually and represents the substitution of proton in the carboxylic acid group during the redox reaction and not in the first reaction cycle. In addition, the redox reaction of MA does not affect the SP.

**Figure 3:**
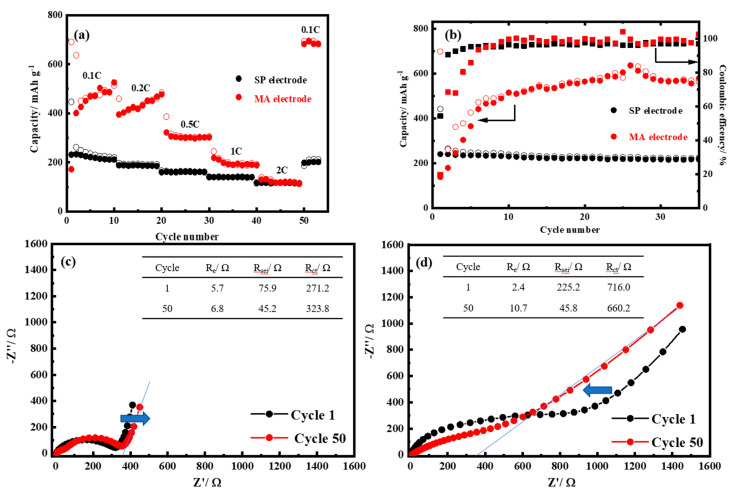
(**a**) The rate capability and (**b**) the cyclability of the SP and the MA electrodes. The electrochemical impedance spectroscopy (EIS) measurements of (**c**) the SP and (**d**) the MA electrodes at the first and the 50th cycle.

To estimate the power and cycle performances of the SP and the MA electrodes, all electrodes were assessed at various rates from 0.1 to 2 C. Figure 3a shows that the SP electrode has an initial capacity of 231.7, 188.3, 161.1, 140.0, and 115.0 mAh g^−1^ at 0.1, 0.2, 0.5, 1, and 2 C, respectively. When the rate decreases to 0.1 C after 50 cycles, the SP electrode reverts to its original capacity to 198.3 mAh g^−1^, being slightly lower than that in the beginning. By contrast, the MA electrode had an initial capacity of 171.9, 394.8, 321.9, 218.8, and 130.2 mAh g^−1^ at 0.1, 0.2, 0.5, 1, and 2 C. On decreasing the rate to 0.1 C after 50 cycles, the discharge capacity at 0.1 C reverted to 685.4 mAh g^−1^. As shown in Figure 3a, the redox reaction of MA at the MA electrode was completed after 20 cycles and stabilized after 0.5 C. A discharging current is applied to accelerate the MA redox reaction. These results indicate that the redox reaction and a small bandgap effect of MA do not affect the cycle performance but significantly improve the power ability. In particular, the SP electrode had a stable capacity of approximately 227.8 mAh g^−1^. The exact capacity of MA was therefore 571.5 mAh g^−1^, corresponding to approximately 2.5 lithium ions being involved in the redox reaction at the MA electrode. The MA has superior cyclability and rate potential than those of previously reported aliphatic and aromatic compounds [26,38,39]. At the MA electrode, the effect of hydrogen on carbonyl groups of the MA increased the capacity [30,40] and the -CONH_2_ group decreased the voltage plateau [15]. In Figure 3b, the cyclability results show that the MA electrode reveals advanced reversible capacity of 585 mAh g^-1^ compared with the SP electrode (220 mAh g^-1^) after 35 cycles at a current rate of 0.1 C. Interestingly, this result demonstrates that the MA needs a redox reaction process for its structure reassembly on the first ten cycles and further delivers the maximum capacity. This behavior confirms the result in Figure 3a.

Figure 3c,d shows the results of EIS analysis at the SP and the MA electrodes. The equivalent circuit represents the electrolyte (R_e_), SEI (R_SEI_), charge-transfer (R_ct_), and the Warburg (W) resistances [32]. The simulated impedance parameters were determined by fitting the original EIS spectra using ZView software (inset). As shown in Figure 3c, the SP electrode has approximately one semicircle, which changes to two semicircles after 50 cycles. SEI formation drastically increased the interface impedance and dramatically changed through subsequent cycles. Simulation results indicated that an increase in R_ct_ (from 271.2 Ω to 323.8 Ω) exerts most of its effects during electrochemical cycling. Although R_e_ decreased, the two semicircles were clearly in accordance with the formation of the incompatible interface. Furthermore, the Warburg slope displayed more prominent behavior beyond 45°, representing a capacitance effect of charge accumulation on the SP surface and almost the same behavior after 50 cycles. Interestingly, the MA electrode displayed different behavior after cycling in Figure 3d. Initially, the semicircle indicated that the R_SEI_ (225.2 Ω) and R_ct_ (716.2 Ω) were markedly higher than those of the SP electrode owing to the redox reaction of the MA and the combinatorial effect of the SEI. After 50 cycles, both R_SEI_ (45.8 Ω) and R_ct_ (660.2 Ω) decreased, accompanied by a significant reduction in the Warburg value (361.4 Ω) in comparison with the SP electrode (383.7 Ω), and a resistance effect was observed. Furthermore, the two semicircles were not prominent in the spectrum, indicating effective cooperation between MA and SP.

**Figure 4:**
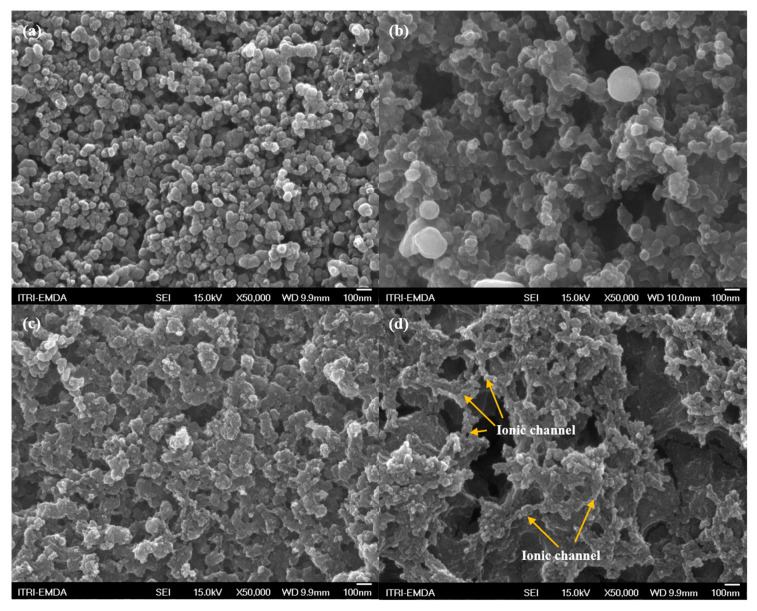
The SEM images of the SP and the MA electrodes (**a**,**b**) before charge and (**c**,**d**) after 50 cycles.

Figure 4 shows the results of SEM at the two electrodes. As shown in Figure 4a, the particle size of SP was approximately 50–100 nm, which is homogeneously dispersed at the electrode. At the MA electrode, the SP was surrounded by the soft matter, MA, and displayed a continuous, uninterrupted ion channel after the electrochemical reaction (Figure 4b). After 50 cycles, the SP expanded owing to repeated lithiation and de-lithiation reactions (Figure 4c). The SEI was prominent on the SP surface and presented an aggregated pattern among particles. However, Figure 4d shows the MA was regenerated during the electrochemical reaction, and the morphology of the MA electrode was altered. Single SP particles were not found, and there was a prominent high ionic channel, indicated by the arrow shown in Figure 4d. These results confirm the marked power contribution and lower Warburg impedance of the MA in Figure 3d.

**Figure 5:**
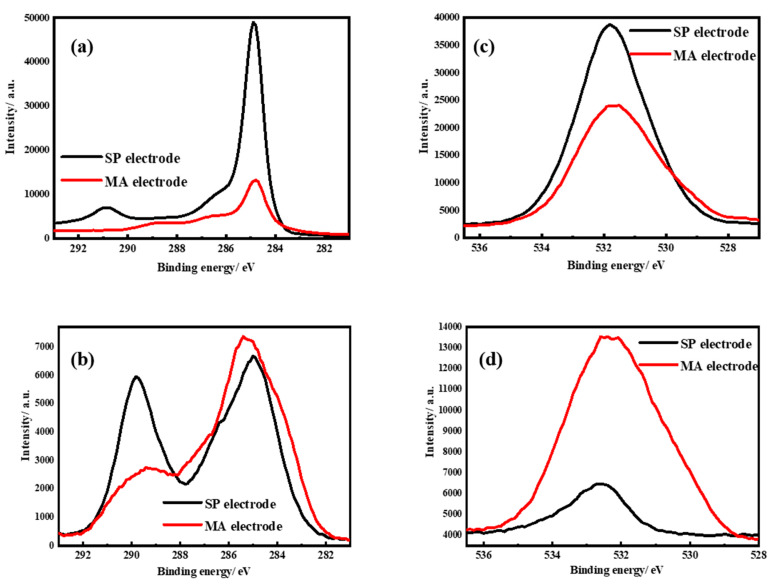
The XPS of C1s spectra of two electrodes (**a**) before charge and (**b**) after 50 cycles. The O1s spectra of two electrodes (**c**) before charge and (**d**) after 50 cycles.

**Scheme 1:**
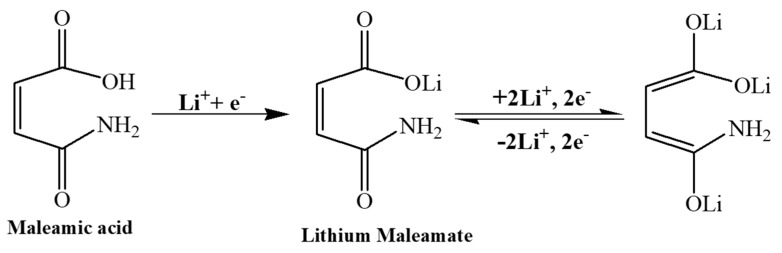
The representative of the lithium-ion storage mechanism on the MA material.

Figure 5 shows the results of the XPS analysis of two electrodes in terms of C1s, O1s, and F1s spectra. Based on previous reports [41,42], Figure 5a,b can be divided into several regions. The 284–286 eV region represents the sp2-hybridized carbon structure at 284.4 eV. Furthermore, the 286–292 eV region represents –C–O–C bonding in RCH_2_OCO_2_Li (alkyl lithium carbonates) at 288 eV, –C–O– bonding in Li_2_CO_3_ at 288.5 eV, and –C=O– bonding in RCH_2_OCO_2_Li at 289.1 eV. Thus, sp2-hybridized carbon is the major constituent of the SP electrode. Furthermore, the MA contains carbonyl and –C–O groups, representing a higher intensity of approximately 285–290 eV in comparison with the SP electrode. After 50 cycles, the SP electrode displayed significant formation of alkyl lithium carbonates and lithium carbonate in the 289–291 eV region, indicating electrolyte decomposition and the SEI formation. However, the SEI formation was not enhanced by MA at the MA electrode, thus markedly inhibiting electrolyte decomposition. Figure 5c,d displays a 527–536 eV region representing Li_2_O at 527 eV, Li_2_CO_3_ or a –C–O–C bonding of RCH_2_OCO_2_Li at 531.5 eV and a –C=O– bonding of RCH_2_OCO_2_Li at 532.5 eV [43]. As shown in Figure 5c, the SP electrode did not contain any such components owing to its carbon structure before the electrochemical reaction. As shown in Figure 5c, the oxygen content increased at the MA electrode owing to the MA involvement. This spectrum displays clear C–O–C and –C=O– bonds in specific regions. After 50 cycles, the SEI was formed at the SP electrode from the electrolyte and comprised a large amount of Li_2_CO_3_ and RCH_2_OCO_2_Li on the SP surface, thus reducing the intensity of sp2-hybridized carbon. Furthermore, Li_2_O was formed at low levels, thus potentially accounting for the increase in charge transfer resistance (Figure 3c). As shown in Figure 5d, a limited increase in the intensity of the SEI was observed at the MA electrode, and no changes were observed in RCH_2_OCO_2_Li levels in comparison with the SP electrode. Moreover, the content of undissociated lithium salt almost remained unchanged at the SP electrode, indicating that the electrolyte is unaffected by MA, with no different reactions on the surface. XPS revealed that the MA electrode containing MA undergoes the same electrochemical reaction and generates the same products on its surface; however, MA adequately inhibits LiF formation. These results suggest that MA stores lithium ions through its carbonyl groups and do not directly react with SP, thus effectively reducing the decomposition and activation of carbonate solvents on the carbon-based anodes [34]. Scheme 1 illustrates a potential mechanism underlying the reaction of MA occurring at the MA electrode. The MA precursor is activated through the redox reaction of lithium ions on its carbonyl group to form lithium maleamate. However, this phenomenon involves gradual electrochemical reactions, probably requiring more than 20 cycles. As shown in Figure 3a, this rearrangement phenomenon may be accelerated at a high current. The morphology of MA in the MA electrode is altered upon completion of the rearrangement phenomenon. Figure 4d shows a prominent soft material with continuous ionic channels. During this process, LiPF_6_ is not formed simultaneously with the SEI on the appearance of graphite, thus reducing lithium carbonate, and alkyl lithium carbonate contents, as revealed through XPS and EIS analyses. Lithium maleamate is then doped and de-doped with lithium ions on further electrochemical cycling.

## 4. Conclusions

This study shows that MA has excellent redox properties and is potentially applicable as a material for mixing or single anode electrodes in LIBs. MA has a high specific capacity, high rate capability, and outstanding cycle performance at various current rates. At the MA electrode, the MA significantly inhibited the formation of the SEI, which significantly reduces the interface impedance. The redox reaction of the MA is harmless and effectively fabricates continuous channels for ionic transfer. MA is an excellent organic material, serving as an appropriate candidate for a modified or single anode material for energy storage.

## References

[1] Dunn B., Kamath H., Tarascon J.-M. (2011). Electrical energy storage for the grid: A battery of choices. Science.

[2] Goodenough J.B., Park K.-S. (2013). The Li-ion rechargeable battery: A perspective. J. Am. Chem. Soc..

[3] Moriwake H., Kuwabara A., Fisher C.A., Ikuhara Y. (2017). Why is sodium-intercalated graphite unstable?. RSC Adv..

[4] Cheng X.-B., Zhang R., Zhao C.-Z., Zhang Q. (2017). Toward safe lithium metal anode in rechargeable batteries: A review. Chem. Rev..

[5] Kahsay B.A., Ramar A., Wang F.-M., Yeh N.-H., Lin P.-L., Luo Z.-J., Chan T.-S., Su C.-H. (2019). Investigating an all-organic battery using polyisothianaphthene as a redox-active bipolar electrode material. J. Power Sources.

[6] Muench S., Wild A., Friebe C., Häupler B., Janoschka T., Schubert U.S. (2016). Polymer-Based Organic Batteries. Chem. Rev..

[7] Zheng T., Jia Z., Lin N., Langer T., Lux S., Lund I., Gentschev A.-C., Qiao J., Liu G. (2017). Molecular spring enabled high-performance anode for lithium ion batteries. Polymers.

[8] Wu M., Cui Y., Bhargav A., Losovyj Y., Siegel A., Agarwal M., Ma Y., Fu Y. (2016). Organotrisulfide: A high capacity cathode material for rechargeable lithium batteries. Angew. Chem. Int. Ed..

[9] Nakahara K., Iwasa S., Satoh M., Morioka Y., Iriyama J., Suguro M., Hasegawa E. (2002). Rechargeable batteries with organic radical cathodes. Chem. Phys. Lett..

[10] Song Z., Zhou H. (2013). Towards sustainable and versatile energy storage devices: An overview of organic electrode materials. Energy Environ. Sci..

[11] Zhang Y.Y., Sun Y.Y., Du S.X., Gao H.J., Zhang S.B. (2012). Organic salts as super-high rate capability materials for lithium-ion batteries. Appl. Phys. Lett..

[12] Luo C., Borodin O., Ji X., Hou S., Gaskell K.J., Fan X., Chen J., Deng T., Wang R., Jiang J. (2018). Azo compounds as a family of organic electrode materials for alkali-ion batteries. Proc. Natl. Acad. Sci. USA.

[13] Amin K., Mao L., Wei Z. (2019). Recent Progress in Polymeric Carbonyl-Based Electrode Materials for Lithium and Sodium Ion Batteries. Macromol. Rapid Commun..

[14] Lyu H., Jafta C.J., Popovs I., Meyer H.M., Hachtel J.A., Huang J., Sumpter B.G., Dai S., Sun X.-G. (2019). A dicyanobenzoquinone based cathode material for rechargeable lithium and sodium ion batteries. J. Mater. Chem. A.

[15] Lu Y., Zhang Q., Li L., Niu Z., Chen J. (2018). Design strategies toward enhancing the performance of organic electrode materials in metal-ion batteries. Chem.

[16] Zhao Q., Lu Y., Chen J. (2017). Advanced Organic Electrode Materials for Rechargeable Sodium-Ion Batteries. Adv. Energy Mater..

[17] Häupler B., Wild A., Schubert U.S. (2015). Carbonyls: Powerful Organic Materials for Secondary Batteries. Adv. Energy Mater..

[18] Sharma P., Damien D., Nagarajan K., Shaijumon M.M., Hariharan M. (2013). Perylene-polyimide-based organic electrode materials for rechargeable lithium batteries. J. Phys. Chem. Lett..

[19] Guo C., Zhang K., Zhao Q., Pei L., Chen J. (2015). High-performance sodium batteries with the 9,10-anthraquinone/CMK-3 cathode and an ether-based electrolyte. ChemComm.

[20] Huang W., Zhu Z., Wang L., Wang S., Li H., Tao Z., Shi J., Guan L., Chen J. (2013). Quasi-Solid-State Rechargeable Lithium-Ion Batteries with a Calix [4] quinone Cathode and Gel Polymer Electrolyte. Angew. Chem. Int. Ed..

[21] Armand M., Grugeon S., Vezin H., Laruelle S., Ribière P., Poizot P., Tarascon J.M. (2009). Conjugated dicarboxylate anodes for Li-ion batteries. Nat. Mater..

[22] Lyu H., Liu J., Mahurin S., Dai S., Guo Z., Sun X.-G. (2017). Polythiophene coated aromatic polyimide enabled ultrafast and sustainable lithium ion batteries. J. Mater. Chem. A.

[23] Siwal S.S., Zhang Q., Devi N., Thakur V.K. (2020). Carbon-based polymer nanocomposite for high-performance energy storage applications. Polymers.

[24] Lyu H., Li P., Liu J., Mahurin S., Chen J., Hensley D.K., Veith G.M., Guo Z., Dai S., Sun X.G. (2018). Aromatic polyimide/graphene composite organic cathodes for fast and sustainable lithium-ion batteries. ChemSusChem.

[25] Rodríguez-Pérez I.A., Yuan Y., Bommier C., Wang X., Ma L., Leonard D.P., Lerner M.M., Carter R.G., Wu T., Greaney P.A. (2017). Mg-Ion Battery Electrode: An Organic Solid’s Herringbone Structure Squeezed upon Mg-Ion Insertion. J. Am. Chem. Soc..

[26] Fédèle L., Sauvage F., Bois J., Tarascon J.-M., Bécuwe M. (2014). Lithium insertion/de-insertion properties of π-extended naphthyl-based dicarboxylate electrode synthesized by freeze-drying. J. Electrochem..

[27] Chen H., Armand M., Demailly G., Dolhem F., Poizot P., Tarascon J.M. (2008). From biomass to a renewable LixC6O6 organic electrode for sustainable Li-ion batteries. ChemSusChem.

[28] Walker W., Grugeon S., Vezin H., Laruelle S., Armand M., Tarascon J.M., Wudl F. (2010). The effect of length and cis/trans relationship of conjugated pathway on secondary battery performance in organolithium electrodes. Electrochem. Commun..

[29] Zhao Q., Guo C., Lu Y., Liu L., Liang J., Chen J. (2016). Rechargeable Lithium Batteries with Electrodes of Small Organic Carbonyl Salts and Advanced Electrolytes. Ind. Eng. Chem..

[30] Wang Y., Deng Y., Qu Q., Zheng X., Zhang J., Liu G., Battaglia V.S., Zheng H. (2017). Ultrahigh-capacity organic anode with high-rate capability and long cycle life for lithium-ion batteries. ACS Energy Lett..

[31] Song Z., Qian Y., Liu X., Zhang T., Zhu Y., Yu H., Otani M., Zhou H. (2014). A quinone-based oligomeric lithium salt for superior Li–organic batteries. Energy Environ. Sci..

[32] Cui H., Li Q., Qiu G., Wang J. (2018). Carbon-chain inserting effect on electronic behavior of single-walled carbon nanotubes: A density functional theory study. MRS Commun..

[33] Peng B., Xu Y., Wang X., Shi X., Mulder F.M. (2017). The electrochemical performance of super P carbon black in reversible Li/Na ion uptake. Sci. China Phys. Mech..

[34] An S.J., Li J., Daniel C., Mohanty D., Nagpure S., Wood D.L. (2016). The state of understanding of the lithium-ion-battery graphite solid electrolyte interphase (SEI) and its relationship to formation cycling. Carbon.

[35] Goriparti S., Harish M.N.K., Sampath S. (2013). Ellagic acid—A novel organic electrode material for high capacity lithium ion batteries. ChemComm.

[36] Abouimrane A., Weng W., Eltayeb H., Cui Y., Niklas J., Poluektov O., Amine K. (2012). Sodium insertion in carboxylate based materials and their application in 3.6 V full sodium cells. Energy Environ. Sci..

[37] Deng Q., Xue J., Zou W., Wang L., Zhou A., Li J. (2016). The electrochemical behaviors of Li_2_C_8_H_4_O_6_ and its corresponding organic acid C_8_H_6_O_6_ as anodes for Li-ion batteries. J. Electroanal. Chem..

[38] Wang S., Wang L., Zhang K., Zhu Z., Tao Z., Chen J. (2013). Organic Li_4_C_8_H_2_O_6_ nanosheets for lithium-ion batteries. Nano Lett..

[39] Zhao R.R., Cao Y.L., Ai X.P., Yang H.X. (2013). Reversible Li and Na storage behaviors of perylenetetracarboxylates as organic anodes for Li- and Na-ion batteries. J. Electroanal. Chem..

[40] Chen L., Liu S., Zhao L., Zhao Y. (2017). OH-substituted 2, 3-dichloro-5, 6-dicyano-1, 4-benzoquinone as highly stable organic electrode for lithium ion battery. Electrochim. Acta.

[41] Światowska J., Lair V., Pereira-Nabais C., Cote G., Marcus P., Chagnes A. (2011). XPS, XRD and SEM characterization of a thin ceria layer deposited onto graphite electrode for application in lithium-ion batteries. Appl. Surf..

[42] Zhao L., Watanabe I., Doi T., Okada S., Yamaki J.-I. (2006). TG-MS analysis of solid electrolyte interphase (SEI) on graphite negative-electrode in lithium-ion batteries. J. Power Sources..

[43] Dedryvère R., Leroy S., Martinez H., Blanchard F., Lemordant D., Gonbeau D. (2006). XPS valence characterization of lithium salts as a tool to study electrode/electrolyte interfaces of Li-ion batteries. J. Phys. Chem. B.

